# Molecular characterization of echovirus 9 strains isolated from hand-foot-and-mouth disease in Kunming, Yunnan Province, China

**DOI:** 10.1038/s41598-022-06309-1

**Published:** 2022-02-10

**Authors:** Ming Zhang, Wei Guo, Danhan Xu, Changzeng Feng, Guohong Bao, Hao Sun, Zhaoqing Yang, Shaohui Ma

**Affiliations:** 1 Institute of Medical Biology, Peking Union Medical College, Chinese Academy of Medical Sciences, Kunming, 650118 People’s Republic of China; 2 Yunnan Key Laboratory of Vaccine Research Development on Severe Infectious Disease, Kunming, 650118 People’s Republic of China; 3 First People’s Hospital of Yunnan Province, Kunming, People’s Republic of China

**Keywords:** Pathogens, Virology

## Abstract

Echovirus 9 (E9) belongs to the species *Enterovirus B*. So far, 12 whole genome sequences of E9 are available in GenBank. In this study, we determined the whole genomic sequences of five E9 strains isolated from the stools of patients with hand-foot-and-mouth disease in Kunming, Yunnan Province, China, in 2019. Their nucleotide and amino acid sequences shared 80.8–80.9% and 96.4–96.8% identity with the prototype Hill strain, respectively, and shared 99.3–99.9% and 99.1–99.8% mutual identity, respectively. Recombination analyses revealed that intertype recombination had occurred in the *2C* and *3D* regions of the five Yunnan E9 strains with coxsackieviruses B5 and B4, respectively. This study augmented the whole genome sequences of E9 in the GenBank database and extended the molecular characterization of this virus in China.

## Introduction

The genus *Enterovirus*, in the family *Picornaviridae*, is further divided into 15 species: *Enterovirus A–K* and *Rhinovirus A–C*, comprising 327 serotypes (https://www.picornaviridae.com). *Enterovirus B* (EV-B) currently consists of 63 serotypes: coxsackieviruses B (CVB1–6), CVA9,echoviruses (E1–7, 9, 11–21, 24–27, 29–33), and enteroviruses B (EV-B69, 73–75, 77–88, 93, 97–101, 106–107, 110–114) (https://www.picornaviridae.com). Like other enteroviruses (EVs), echoviruses are small nonenveloped, single-stranded positive-sense RNA viruses. The echovirus genomes are about 7400 nucleotides long and contain a long open reading frame (ORF), which encodes a polyprotein that is processed into three polyprotein precursors (*P1*, *P2*, and *P3*). These are further cleaved into four structural proteins (*VP4*, *VP2*, *VP3*, and *VP1*) and seven nonstructural proteins (*2A*, *2B*, *2C*, *3A*, *3B*, *3C*, and *3D*). The ORF is flanked by a 5′-untranslated region (UTR) and a 3′-UTR^1,2^.

EVs are commonly associated with asymptomatic or mild infections, such as fever, irritation, and hand-foot-and-mouth disease (HFMD), although some patients may develop severe diseases, including aseptic meningitis and acute flaccid paralysis. EVs are the main causative pathogen of aseptic meningitis^3–5^. Among the EVs, E9 is often associated with aseptic meningitis^6–13^ and HFMD^14–18^, and has been detected in a case of herpangina^19,20^. Previous studies have reported that E9 was main causative pathogen of aseptic meningitis and HFMD in Yunnan, China, in 2009–2010^13,14^. Twelve whole genome sequences of E9 are currently available in GenBank, which were isolated from children with aseptic meningitis or HFMD, healthy children, and children with other diseases. However, only one is from a Chinese strain. In this study, the whole genomic sequences of five E9 strains recovered from children aged 0.9–4.5 years with HFMD in Kunming, Yunnan Province, China, in 2019 were determined to clarify the molecular characteristics of E9 in China and to increase the number of genome sequences of E9 in the GenBank database.

## Results

### Primary characterization and whole-VP1 sequence analysis

Four E9 strains (115K3/YN/CHN/2019 [115K3], 123K3/YN/CHN/2019 [123K3], 133K3/YN/CHN/2013 [133K3], and 121K3/YN/CHN/2019 [121K3]) were recovered from human embryonic lung diploid fibroblasts (KMB17) cells, and one strain (115V3/YN/CHN/2019 [115V3]) was recovered from Vero cells, whereas none were from human rhabdomyosarcoma (RD) cells. Of these isolates, 115K3 and 115V3 were isolated from the same sample but from KMB17 and Vero cells rather than human rhabdomyosarcoma (RD) cells. They were isolated from three boys and one girl, ranging in age from 0.9 to 4.5 years.

The whole *VP1* sequences (918 nucleotides) of the five Yunnan strains showed the greatest identity (94.4%–94.8%) with E9 strain Echo9/FJPT176/CHN/2016 (MG922545), isolated from a patient with HFMD in China. The five isolates shared 78.3%–78.5% nucleotide identity and 85.7%–86.4% amino acid identity with the whole *VP1* sequence of the E9 prototype Hill strain, which was isolated from a healthy child in Cincinnati in 1953^21^, and 80.8%–94.8% nucleotide and 88.1%–99.6% amino acid identity with other E9 strains. The whole-*VP1* nucleotide and amino acid identities among the five Yunnan isolates were 99.6%–99.9% and 99.3%–100%, respectively.

**Figure 1:**
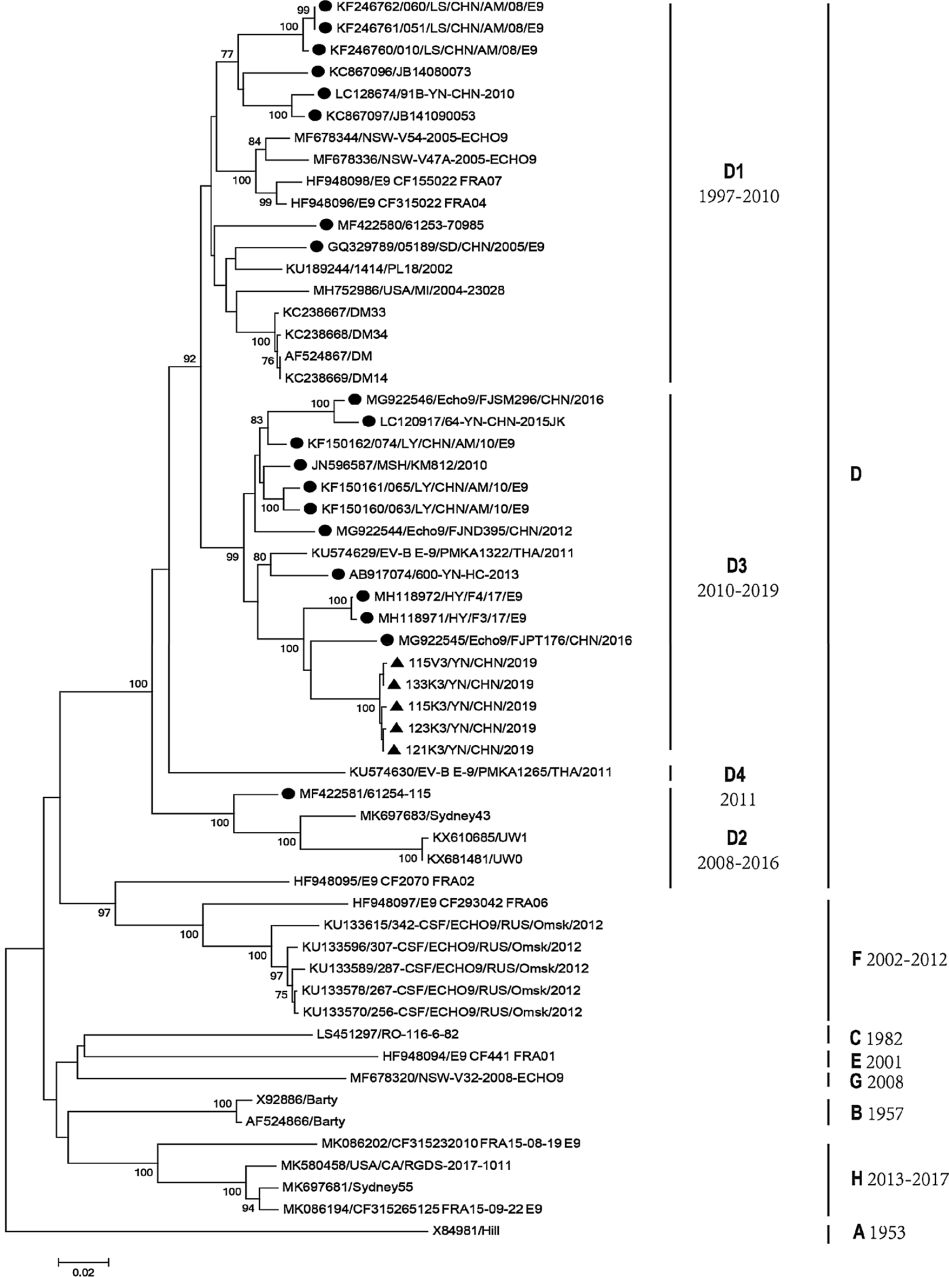
Phylogenetic tree based on whole *VP1* sequences (918 bp) of global E9 isolates. Squares indicate the E9 isolates used in the present study, and triangles indicate other Chinese isolates. Filled triangle: strains isolated in this investigation. Filled circle: other Chinese isolates.

The 53 whole *VP1* sequences available in GenBank were included in an analysis of the five isolates collected in this study (Fig. 1)**.** According to the approximate mean 15% cutoff divergence value used to genotype enterovirus A71 (EV-A71)^22^, the E9 strains were divided into eight clusters (A–H). The main epidemic strains belonged to the D, F, and H clusters. Of these, cluster D contained most E9 strains (40 strains), including all the Chinese strains, together with isolates from Poland, Belgium, Australia, France, the USA, and Thailand, collected in 1997–2019. Cluster F included seven strains from France and Russian, collected in 2002–2012. Cluster H included four strains from the USA, Australia, and France, collected in 2013–2017.

### Selection pressure analysis of the E9 VP1 gene

For the VP1 gene, no positive selection sites were found in the E9 Yunnan strains. However, only one negative selection site was found at amino acid position 286 of VP1 (position 286, H → R) (Table 1).

**Table 1:** Echovirus 9 VP1 negative selection site.

Position aa	Method							
	SLAC		FEL		MEME		FUBAR	
	dN/dS	p-value	dN/dS	p-value	ω +	p-value	dN/dS	Post. Pr
286	0.082	0.009	150.338	0.000	170.18	0.06	−2.43	0.994

### Whole-genome sequence analysis

The whole genome sequences of the five strains (115K3, 115V3, 123K3, 133K3, and 121K3) isolated in Yunnan Province in 2019 were determined. The genome sequences were 7445–7450 nucleotides in length, containing an ORF of 6612 nucleotides, which encoded a polyprotein of 2203 amino acids. The ORF sequence was flanked by a noncoding 5′-UTR of 733–738 nucleotides and a noncoding 3′-UTR of 103–106 nucleotides. The whole-genome nucleotide and amino acid identities of the five isolates were 99.3–99.9% and 99.1–99.8%, respectively. The total base compositions were 28.2–28.4% A, 24.6–24.7% G, 23.1–23.2% C, and 23.9–24.1% U. Because the mutual identities of the whole-genome nucleotide and deduced amino acid sequences of the five strains were > 99.1%, strain 115V3 was selected as the representative strain for further analysis.

Pairwise comparisons of the nucleotide and amino acid sequences of strain 115V3 and the E9 prototype Hill strain and other E9 strains are shown in Table 2. Strain 115V3 shares 79.0% and 80.5–88.7% nucleotide identities with the whole genomes of the E9 prototype Hill strain and the other E9 strains, respectively, and derived amino acid sequence identities of 94.4% and 93.5–97.6%, respectively.

**Table 2:** Nucleotide and amino acid sequence identity between 115V3/YN/CHN/2019 and the prototype E9 and other prototype echovirus strains in all sequenced genomic regions.

Genomic region	Prototype echovirus 9		Other echovirus 9	
	% nucleotide identity	% amino acid identity	% nucleotide identity	% amino acid identity
5′-UTR	77.9		83.7–94.0	
VP4	83.1	94.2	74.9–93.2	91.3–98.6
VP2	80.7	96.6	82.5–94.1	88.1–99.6
VP3	79.9	96.7	76.2–91.8	90.4–99.2
VP1	80.7	96.3	75.1–94.3	88.1–99.6
2A	78.8	93.8	78.0–92.1	91.0–95.8
2B	78.9	92.9	77.2–79.9	90.9–94.9
2C	80.0	97.3	80.4–86.7	96.6–98.8
3A	77.9	95.5	79.4–84.6	95.5–98.9
3B	77.3	95.5	75.8–84.8	84.6–90.9
3C	78.1	96.7	79.6–85.6	95.6–98.1
3D	79.2	96.8	79.2–85.0	96.3–98.1
3′-UTR	83.5		68.9–84.9	
Genome	79.0	94.4	80.5–88.7	93.5–97.6

### Phylogenetic analysis of *P1*, *P2*, and *P3* regions

**Figure 2:**
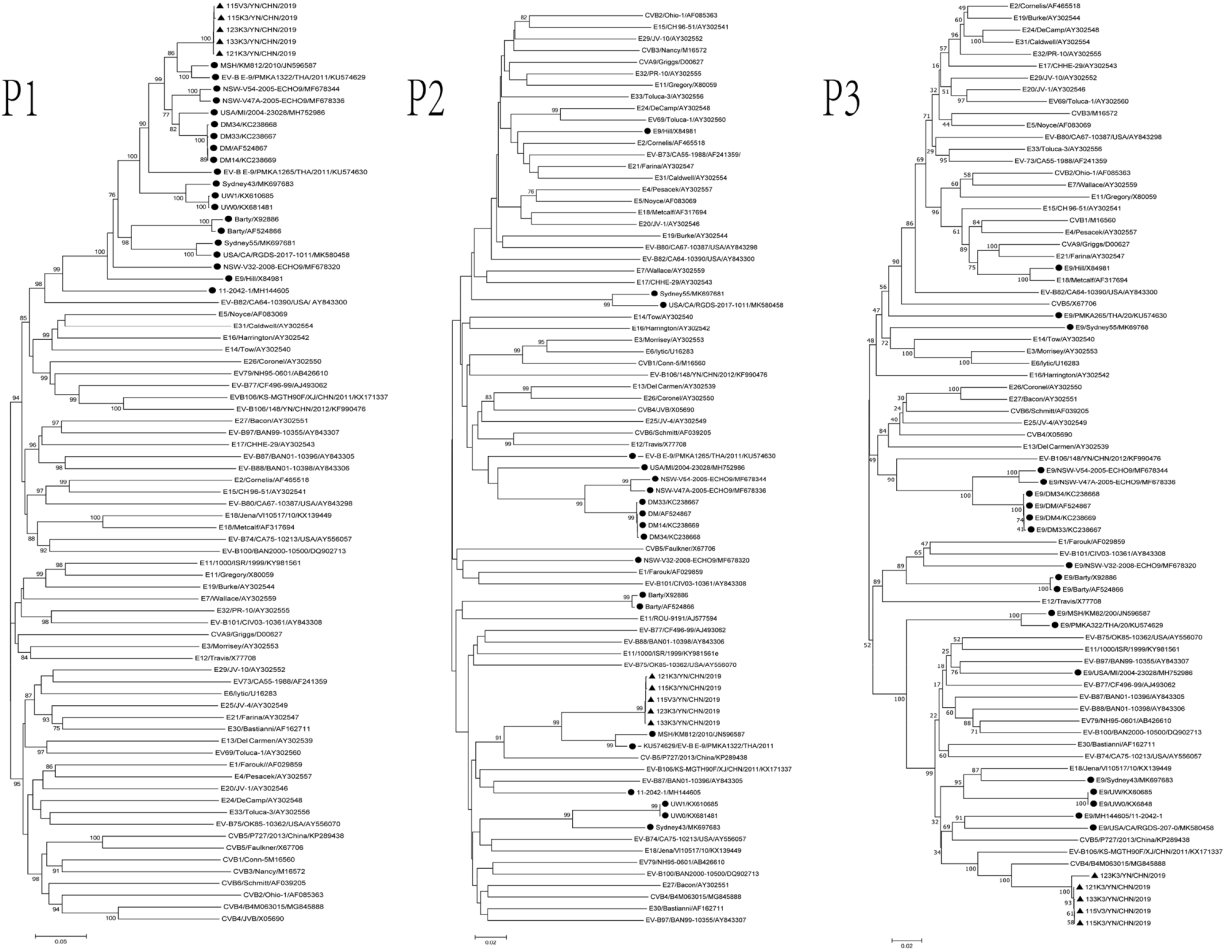
Phylogenetic relationships based on the *P1*, *P2*, and *P3* coding sequences of all E9 strains, all EV-B prototype strains available in the GenBank database, the five Yunnan isolates, and three EV-B strains (CV-B5/P727/2013/China, CVB4-B4M063015 and E11-1000/ISR/1999), as analyzed by nucleotide sequence alignment with the neighbor joining algorithms implemented in the MEGA 6.06 program. Numbers at nodes indicate bootstrap support for that node (percentage of 1000 bootstrap replicates). The scale bars represent the genetic distance. Only high bootstrap values (> 75%) are shown. Filled triangle: strains isolated in this investigation. Filled circle: E9 strains.

Phylogenetic trees were constructed for *P1*, *P2*, and *P3* regions of all E9 strains and EV-B prototype strains available in GenBank, the five Yunnan isolates, and three EV-B strains (CV-B5/P727/2013/China, CVB4-B4M063015, and E11-1000/ISR/1999) (Fig. 2). In the *P1* region, the Yunnan isolates grouped together with all E9 strains, including the E9 prototype Hill strain, thus further confirming the initial typing results. However, in the P2 and P3 coding regions, the E9 strains clustered with different EV-B strains and formed different clusters (Fig. 2). In the *P2* region, the Yunnan isolates clustered with two E9 isolates (MSH/KM812/2010 and E-9/PMKA1322/THA/2011) and one CV-B5 strain, CV-B5/P727/2013/China (KP289438). In the *P3* region, the Yunnan isolates clustered with one CVB4 strain, B4M063015 (MG845888). The prototype Hill strain formed a lineage with th**e** E18 prototype strain Metcalf (AF317694), which is a recombinant with the Hill strain^23^. The results indicated that several potential recombination events have occurred between the E9 strains, including the Yunnan isolates, and other EV-B strains in the non-capsid coding regions, and that these E9 isolates may be the different recombinant strains.

### Recombination analysis

The sequences sharing the greatest identity with strain 115V3 were screened online with BLAST (Table 3). The *P3* region of strain 115V3 shared greatest identity with that of CVB4 strain B4 M063015, whereas in the *2C* and *3A* regions, it shared the greatest identity (90.47%–91.43%) with the CVB5 strain P727/2013/China, and in the *3B*, *3C*, and *3D* regions, it shared the greatest identity (89.39%–94.16%) with CVB4 strain B4M063015. In the 3′-UTR, strain 115V3 shared the greatest identity (92.93%) with E11 strain 1000/ISR/1999.

**Table 3:** Highest similarity of nucleotide sequences of enteroviruses in all sequenced genomic regions of the 115V3/YN/CHN/2019 strain, determined with BLAST online.

Genomic region	115V3/YN/CHN/2019				
	Type	Strain	% nucleotide identity	Accession number	Disease
5-UTR	E9	MSH/KM812/2010	94.51	JN596587	Hand, foot and mouth disease
VP4	E9	DMKA1322/THA/2011	97.09	KU574629	Influenza-like illness
VP2	E9	MSH/KM812/2010	94.13	JN596587	Hand, foot and mouth disease
VP3	E9	DMKA1322/THA/2011	92.61	KU574629	Influenza-like illness
VP1	E9	FJDT176/CHN/2016	94.66	MG922545	Hand, foot and mouth disease
2A	E9	DMKA1322/THA/2011	95.37	KU574629	Hand, foot and mouth disease
2B	E9	DMKA1322/THA/2011	95.30	KU574629	Hand, foot and mouth disease
2C	CVB5	P727/2013/China	90.47	KP289438	Hand, foot and mouth disease
3A	CVB5	P727/2013/China	91.73	KP289438	Hand, foot and mouth disease
3B	CVB4	B4M063015	89.39	MG845888	Raw sewage
3C	CVB4	B4M063015	93.63	MG845888	Raw sewage
3D	CVB4	B4 M063015	94.16	MG845888	Raw sewage
3-UTR	E11	1000/ISR/1999	92.93	KY981561	Immunodeficient patient
P1	E9	DMKA1322/THA/2011	93.53	KX767786	Influenza-like illness
P2	E9	DMKA1322/THA/2011	90.84	KX767786	Influenza-like illness
P3	CVB4	B4 M063015	92.51	MG845888	Raw sewage

**Figure 3:**
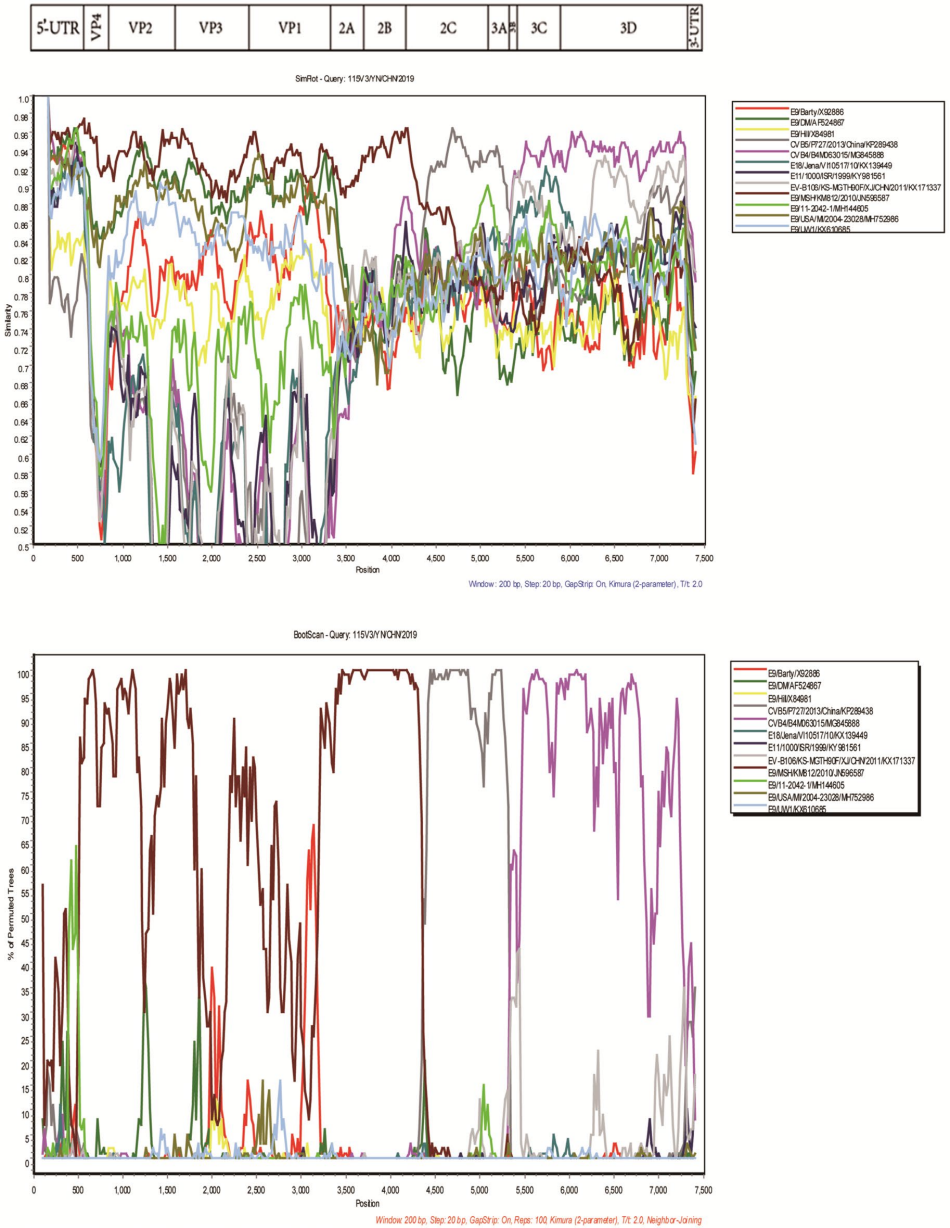
Similarity plot and bootscanning analysis of 115V3 strains with closely related strains. The analyses were conducted via Simplot 3.5.1 with a sliding window of 200 nucleotides moving in steps of 20 nucleotides.

**Figure 4:**
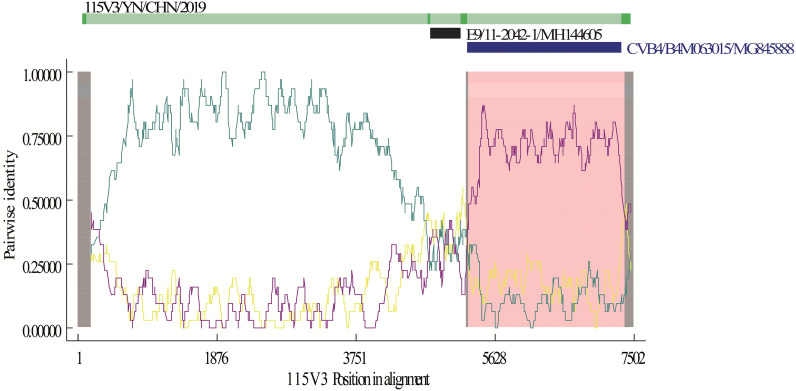
RDP4 software analysis of strain 115V3 with closely related strains. Seven algorithms were used for recombination analysis: RDP, GENECONV, BootScan, Maxchi, Chimaera, SiScan, and 3Seq.

With a similarity plot and a bootscanning analysis, several recombination events were confirmed in the genomic sequence of strain 115V3 (Fig. 3). In the *P1* region, strain 115V3 showed greatest identity (> 90%) with the E9 strain MSH/KM812/2010. However, in the *2C*–*3A* regions and the *3B*–*3D* region, strain 115V3 shared greatest homologies (> 92%) with CVB5 strain P727/2013 and CVB4 strain B4M063015, respectively. Furthermore, RDP4 analysis revealed that the 115V3 isolate underwent a recombination event involving the strain CVB4/B4M063015/MG845888. The recombination event started at approximately 5300 nt and ended at approximately 7350 nt, in the *P3* region of the isolates’ genomes (Fig. 4).

## Discussion

Since 2008, HFMD caused by EVs has become a serious infectious disease in mainland China, mainly occurring in children under 5 years of age. Many EVs, including echoviruses, cocirculated in both sporadic and epidemic cases of HFMD^14–18^. Since the introduction of an inactivated EV-A71 vaccine, the prevalent etiological agents of HFMD have changed, and CV-A6 has become the main pathogen in mainland China^24,25^. EVs, and especially echoviruses, are the major agents of aseptic meningitis^3–5^, and EV-B, including echoviruses, can also cause HFMD^18–20^. The serotypes of the EV pathogens causing HFMD are the same as those causing aseptic meningitis. Therefore, the persistent surveillance of the pathogens responsible for HFMD and aseptic meningitis is very important, and may allow the main serotypes of EVs associated with outbreaks to be predicted.

Because of their error-prone replication, EVs form highly polymorphic populations within their hosts. Since the prototype Hill strain was isolated in 1953^21^ and the Barty strain was isolated from a child with aseptic meningitis in 1957^26^, E9 isolates have evolved into eight clusters, thus indicating that E9 is genetically diverse. On the basis of analysis of the entire VP1 gene, the Chinese E9 strains were divided into D1 (2008–2010) and D3 (2010–2019) clusters, and the five Yunnan isolates in the study formed a single cluster. Furthermore, the average VP1 nucleotide and amino acid sequence divergence was 8.3% (5.3–11.2%) and 2.75% (0.3–5.2%) between the five Yunnan isolates and all whole VP1 gene sequences of other Chinese strains available in GenBank, respectively. In particular, the only whole genome of the Chinese E9 strain available in GenBank, MSH/KM812/2010, which was isolated from a patient with HFMD in 2010 in Yunnan Province, had the nucleotide and amino acid sequence divergence of 12.65% (12.5–12.8%) and 3.1% (2.4–3.8%), respectively. This finding indicated that the Chinese strains have evolved. Therefore, we speculate that the five Yunnan strains have adapted to a changing environment, in response to selection pressures.

The *VP1* capsid protein of the EVs is located together with many immunodominant-serotype-specific epitopes in the exposed B-C loop. Although *VP1* is the most variable of the capsid proteins, the N-terminus of *VP1*, which contains the B-C loop, is highly conserved within individual enteroviral serotypes^27^. The B-C loop is located inside the viral particle and is negligibly influenced by the immune pressure exerted by the host. The sequence of the E9 B-C loop, GDPESTDRFDA (amino acids 83–93 of the *VP1* protein)^28^, in the five Yunnan strains is the same as that in other recent China strains, such as HY/F4/17/E9 (2017, aseptic meningitis), HY/F3/17/E9 (2017, aseptic meningitis), Echo9/FJSM296/CHN/2016 (2016, HFMD), Echo9/FJPT176/CHN/2016 (2016, HFMD), 060/LS/CHN/AM/08/E9 (aseptic meningitis), 051/LS/CHN/AM/08/E9, and 61253–70985 (aseptic meningitis). This indicates that the B-C loop is highly conserved in the Chinese strains. Moreover, amino acid A81, which is exposed at the surface near the B-C loop in the *VP1* protein, is shared by most E9 strains, including the five Yunnan strains. A previous study reported that an E9 strain carrying the T81A substitution in VP1 had lytic potential toward pancreatic cells^28^. However, the non-lytic E9 strains Hill and Barty, including the five Yunnan strains in the study (dada not shown), contain alanine at this site. Thus, additional genetic substitutions in the viral genome may be very important in the pathogenicity toward pancreatic islets, and further research is required^29^.

The RGD motif at the C-terminus of the *VP1* protein is very important to the pathogenicity of E9^30^. The interaction between the virus and the cell occurs via the contact between the RGD motif and the host cell receptor^29^. The Barty strain, which is highly virulent in newborn mice, and other E9 strains, including three of the Yunnan strains, contain this motif, whereas the Hill strain, which is nonpathogenic to newborn mice, does not. This suggests that these three Yunnan strains would display the same pathogenicity as the Barty strain. However, the RGD → GGD substitution is present in strains 115V3 and 133K3, although the five Yunnan strains were all isolated from patients with HFMD, without aseptic meningitis. Therefore, the RGD → GGD substitution requires further study. For EV, *VP1* is the most immunogenic protein and is involved in its host cell with receptor-mediated entry ^31^. However, one negative selection site (*VP1* 286 H → R) was found in the study. We speculate that although the amino acids are basic, this substitution may affect viral function, although this possibility requires further study.

Recombination is a major mechanism of enteroviral evolution*,* particularly that of EV-B^32,33^. The E9 prototype Hill strain is a recombinant between the E9 strain Barty and the E18 prototype strain Metcalf^23^. Therefore, we used BLAST online to screen the strains sharing the highest identity with the E9 strain 115V3 in different regions of the E9 genome. We consequently identified several putative recombination events in different coding regions between strain 115V3 and the CVB5 strain CV-B5-P727/2013/China, the CVB4 strain B4M063015, and the E11 strain 1000/ISR/1999. On the basis of the very high (approximately 90%) sequence identities, we inferred that these viruses are recombination partners. Among them, CBV5 strain CV-B5-P727/2013/China was isolated from patients with HFMD^34^, the CVB4 strain B4M063015 was isolated from raw sewage^35^, and the E11 strain 1000/ISR/1999 was isolated from a chronically infected immunodeficient patient. Es play major roles in natural recombination events among the coxsackieviruses B (CVBs)^36^. CVB infections are associated with HFMD, aseptic meningitis, acute myocarditis, and fatal neonatal infections^37–39^. CVB5 is one of the five most common EVs in the USA^40^, and it has the highest prevalence in Germany and Spain^3,41^. CVB5 was also involved in an outbreak of neurological HFMD in China^42^. The numbers of cases of HFMD caused by CVB4 and CVB5 have been reported to be increasing in China^36^. These viruses also frequently cocirculate in patients with HFMD in China^43^. Therefore, their epidemiological characteristics may offer these viruses sufficient opportunities for mixing and recombination.

In conclusion, the E9 strains were highly genetically diverse, and intertypic recombination events have occurred in the genomic regions encoding nonstructural proteins. This serotype is globally widespread, and frequently cocirculates with other EVBs. Recombination and mutation drive the evolution of E9. Although the five Yunnan E9 strains analyzed in this study were isolated from HFMD patients without aseptic meningitis, other possible pathogenicities, including aseptic meningitis, cannot be ruled out, and the pathogenicity of these strains warrants further study. Systematic epidemiological surveillance is required to assess the links between E9 and its associated diseases.

## Materials and methods

### Ethics statement

The research content of this study was approved by the Institutional Review Boards of the Institute of Medical Biology, Chinese Academy of Medical Sciences & Peking Union Medical College and complied with the ethical regulations of that institution. The experimenters and staff involved in the project gave their informed consent, and informed consent was obtained from the patients’ families. The samples used in this experiment were obtained from stools collected from four children with HFMD, aged 0.9–4.5 years.

### Viral isolation

The viruses were isolated from the samples with three different cell lines (KMB17, Vero, and RD)^44^. These cells were provided by the Chinese Academy of Medical Sciences. Positive cultures (those with cytopathic effects after three passages) were stored at −80 °C.

### Viral identification and sequencing

The culture supernatants were collected and freeze-thawed three times to allow the complete release of the virus from the cells. The viral RNAs were extracted from the culture supernatants with a QIAamp Viral RNA Mini Kit (QIAGEN, CA, USA) according to the instruction manual. Reverse transcription-polymerase chain reaction (RT-PCR)^44^ was performed with a PrimeScript™ One Step RT-PCR Kit Ver.2 (TaKaRa, Dalian, China) with primer pairs AN88 (TACTGGACCACCTGGNGGNA) and AN89 (CCAGCACTGACAGCAGYNG). The PCR-positive products were sequenced with an ABI 3130 Genetic Analyzer (Applied Biosystems, USA) at Kunming Tsingke Biotechnology Co., Ltd. (Kunming, Yunnan Province, China). The sequences were compared with BLAST (http://www.ncbi.nlm.nih.gov/BLAST/).

The serotype was identified by comparison of the nucleotide sequence with known sequences using BLAST^45^. Two long-distance PCR amplifications were performed with a PrimeScript™ One Step RT-PCR Kit Ver.2 (TaKaRa, Dalian, China) and the following primer pairs: E201F (TTAAAACAGCCTGTGGGTTG) and E93R (TCCACATCAAAGCGCAAGTA) for 5´ end amplification, and E93F (AGGCATGTGAAAAATTACCA) and E98R (ACCGAATGCGGAGAATTTAC) for 3’ end amplification. The primers used for sequencing of the whole-length genome were designed by a primer-walking” strategy^46^. All primers used in the study are listed in Table 2. The PCR products were purified with a QIAquick PCR purification kit (Qiagen, Germany), and sequenced in both directions at least twice with an ABI 3130 Genetic Analyzer (Applied Biosystems, USA).

### Selection pressure analysis of the E9 VP1 gene

The selection pressure of E9 VP1 was predicted with the Datamonkey online Application^46^ (http://www.datamonkey.org) and calculated with the following four methods: mixed-effects model of evolution (MEME); fixed-effects likelihood (FEL); fast unbiased Bayesian approximation (FUBAR); and single likelihood ancestor count (SLAC). The significance level was set at 0.05 for MEME, FEL, and SLAC and at 0.9 for FUBAR. Validated results obtained from both methods were considered credible (Table 4).

**Table 4:** Primers used for complete genome amplification and sequencing.

Primer	Sequence (5′ → 3′)	Nucleotide position	Orientation
AN88	TACTGGACCACCTGGNGGNAYRWACAT	2977–2951	Reverse
AN89	CCAGCACTGACAGCAGYNGARAYNGG	2602–2627	Forward
E201F	TTAAAACAGCCTGTGGGTTG	1–20	Forward
E93R	TCCACATCAAAGCGCAAGTA	2805–2786	Reverse
E93F	AGGCATGTGAAAAATTACCA	2600–2619	Forward
E98R	ACCGAATGCGGAGAATTTAC	7444–7426	Reverse
E97f	GGCCTGGCACAATGGAGA	7228–7245	Forward
E96r	ACATCCAACTGCACTGCC	6601–6584	Reverse
E94f	TGCATATGGGAAGATTAC	3440–3457	Forward
E95f	CGCCATCACAGAGCGATC	42,961–4313	Forward
E96f	AGCAACACTCGAGGCACT	5059–5076	Forward
E91331f1	TACCATATAGCTATTGGA	613–630	Forward
E91r	TCAATAGACTCTTCAC	437–414	Reverse
E91f	AATTATTACAAGGATGCAGC	833–852	Forward
E91213f	CCTAGGATGTCCATCCCA	2984–3001	Forward
E96f1	TGTATGGACAAGTATGGC	6395–6412	Forward

### Sequence analysis and recombination analysis

Nucleotide and amino acid sequence analyses was performed with the Geneious Basic 5.4.1 Beta software. MEGA 7.0 was used for the phylogenetic analysis^47^, with a Kimura two-parameter model and the neighbor-joining method, with 1000 bootstrap replicates. The Recombination Detection Program (RDP) package Beta 4.101 was used to identify recombinant sequences in the default mode. Recombination analysis was performed with the seven algorithms in the following programs: RDP, GENECONV, BootScan, Maxchi, Chimaera, SiScan, and 3Seq. Recombination events with significance p < 0.01 obtained by three or more algorithms were considered reliable. The Jukes-Cantor method was used^48^ in Simplot 3.5.1, with each 200-nucleotide (nt) window shifted in 20 nt steps, and a similarity plot was constructed and a bootscanning analysis performed. Strain 115V3/YN/CHN/2019 was compared with other GenBank-listed sequences to identify the sequences that shared the highest homology with 115V3/YN/CHN/2019 in each genomic segment, and the most similar strains for each segment are listed in Table 3.

### Nucleotide sequence accession numbers

The whole genomes of the five E9 strains isolated in this study have been submitted to GenBank under accession numbers MZ488277–MZ488281.
